# Investigation of the Neurotoxic Effects and Mechanisms of Michler’s Ketone as Investigated by Network Toxicology and Transcriptomics

**DOI:** 10.3390/biology15010003

**Published:** 2025-12-19

**Authors:** Jun Hu, Xianke Zha, Xin Liu, Huilin Jin, Yue Fan, Xin Zhao, Jie Hu, Jian Wang

**Affiliations:** 1 Nanjing Institute of Environmental Sciences, Ministry of Ecology and Environment, Nanjing 210042, China; hujun@nies.org (J.H.); 18851419553@163.com (X.L.); 19850866230@163.com (H.J.); 2 Nursing Program, Kangda College of Nanjing Medical University, Lianyungang 222000, China; zxk114514@outlook.com; 3 Jiangsu Key Laboratory of Preventive and Translational Medicine for Geriatric Diseases, Department of Toxicology, School of Public Health, Suzhou Medical College of Soochow University, Suzhou 215123, China; 20235247076@stu.suda.edu.cn (Y.F.); zhaoxinsuda@163.com (X.Z.)

**Keywords:** aromatic ketones, neurotoxicity, calcium signaling, network toxicology

## Abstract

Michler’s Ketone (MK) is widely utilized as an additive in pigments, dyes, and other colorants, and has become a non-negligible environmental presence. Currently, environmental monitoring data and toxicity data for MK are extremely limited, and its specific mechanisms of neurotoxicity remain poorly characterized. A zebrafish model was employed to systematically delineate the neurotoxic mechanisms of MK through the integration of network toxicology predictions, transcriptomic profiling, and RT-qPCR validation. The results demonstrated that MK exposure was found to induce oxidative stress in zebrafish larvae, which subsequently disrupted the calcium signaling pathway and triggered apoptosis, ultimately leading to neurodevelopmental and locomotor behavioral impairments. This study provides a fundamental basis for elucidating MK’s developmental neurotoxicity mechanisms, while also holding significant value for its ecological risk assessment.

## Simple Summary

Michler’s Ketone (MK), an aromatic ketone widely used in pigments, sunscreens, and packaging materials, has unclear developmental neurotoxicity and mechanisms. This study used zebrafish as a model and integrated techniques such as network toxicology, transcriptomics, qPCR, and behavioral analyses to systematically investigate the neurotoxic effects and molecular mechanisms of MK. The research established a comprehensive evidence chain for MK’s neurotoxicity, providing a scientific basis for its aquatic ecological risk assessment and the development of safety standards for related products.

## 1. Introduction

Aromatic ketones are an important class of organic compounds that have garnered ongoing attention from both academic and environmental fields due to their wide range of applications and potential ecological risks. Michler’s Ketone (MK), an electron-rich benzophenone derivative, exhibits excellent light-absorption properties and is widely used as an additive in colorants such as pigments and dyes. Its application history spanned from the 1990s to the early 2000s, extending from sunscreen formulations to packaging materials, with its UV protection and photostability properties remaining central throughout [1].

Currently, environmental monitoring data on Michler’s Ketone remain relatively limited. Existing research indicates that in 15 types of food packaging paperboard, the detected concentration of Michler’s Ketone ranged from 0.6 to 2.5 μg/g. Under conditions using 95% ethanol as a food simulant, migration of Michler’s Ketone from paperboard to food was observed in 13 samples, with migration concentrations ranging from 1.9 to 9.0 μg/L [2]. Another study reported the detection of MK in 9 types of recycled paper/paperboard at concentrations ranging from 1.7 to 12 μg/g, significantly higher than the previously mentioned results. This is primarily because Michler’s Ketone is a key photoinitiator in UV-curable inks. During the pulping process of recycled waste paper (such as newspapers, magazines, and discarded food packaging), residual inks are not completely removed, leading to the presence of Michler’s Ketone in the final products [3]. Hence, in-depth research on the potential impacts of Michler’s Ketone on the ecological environment and human health holds significant practical importance.

In 1979, in vivo studies conducted by the U.S. National Cancer Institute confirmed that Michler’s Ketone is carcinogenic in animals, capable of inducing hepatocellular carcinoma in rats and hemangiosarcoma in mice [4,5]. At the cellular level, in vitro experiments on Chinese hamsters demonstrated that Michler’s Ketone exhibits significant cytotoxicity at a concentration of 1.5 μg/mL, leading to cell division arrest and chromosomal abnormalities [6]. A 2007 comparative metabolic capacity study revealed that the metabolic clearance of Michler’s Ketone in rainbow trout is far lower than that in rat hepatocytes, indicating a higher potential for accumulation in fish and an increased risk of chronic toxicity [7]. Current research has clearly established that Michler’s Ketone can cause dose-dependent DNA damage in the liver [4], which is one of its primary toxic effects. However, current studies have mostly focused on the acute toxicity of MK to organs such as the liver and kidneys, while research on its neurotoxicity remains relatively scarce. Only a limited number of in vitro cell experiments have suggested a potential risk of neuronal damage, but systematic in vivo validation is lacking. Furthermore, its effects on key neurodevelopmental processes and the underlying molecular mechanisms remain unclear.

Zebrafish have been widely adopted as an important model for evaluating the toxicity of environmental pollutants [8,9]. To investigate the neurotoxicity of MK in zebrafish larvae and its mechanisms, the potential toxicity was first predicted using network toxicology methods. Subsequently, transgenic zebrafish lines *Tg(huc:eGFP)* and *Tg(hb9:eGFP)* were utilized to thoroughly evaluate the effects of MK on neural development. Finally, the toxic mechanisms were systematically investigated through integrated transcriptomic analysis and qPCR validation.

This study was designed to systematically predict and validate the neurotoxicity targets and underlying mechanisms of MK through the integration of network toxicology, transcriptomics, and qPCR technologies using zebrafish as the model organism. As a representative aromatic ketone compound, the in-depth investigation of MK is expected not only to reveal its intrinsic toxic mechanisms but also to provide critical scientific basis for establishing safety regulations for related products and conducting environmental risk assessments, thereby holding significant implications for safeguarding ecological security and population health.

## 2. Materials and Methods

### 2.1. Chemicals

Michler’s Ketone (CAS: 90-94-8) was purchased from Shanghai Rhawn Chemical Technology Co., Ltd. (Shanghai, China) TRIzol, reverse reagent kits, and SYBR GREEN RT-PCR kits were purchased from Takara (Takala, Dalian, China). Dimethyl sulfoxide (DMSO, CAS: 67-68-5) was obtained from Beijing Solarbio Science & Technology Co., Ltd. (Beijing, China) PCR primers were purchased from GENEray Biotechnology (Shanghai, China).

### 2.2. Zebrafish Husbandry

All animal experiments were conducted in accordance with the Guidelines for the Care and Use of Laboratory Animals (IACUC20250710) (Figure S1). Four-month-old adult zebrafish (wild-type AB strain and transgenic zebrafish lines [*Tg(huc:eGFP)*] and [*Tg(hb9:eGFP)*]) were purchased from the Institute of Hydrobiology, Chinese Academy of Sciences (Wuhan, China). The zebrafish were maintained in a recirculating system where the water temperature was maintained at 28 ± 0.5 °C and the photoperiod was set at a 14-h light/10-h dark cycle. The fish were fed brine shrimp twice daily. Healthy embryos were selected for experiments after being examined under a microscope.

### 2.3. Construction of Neurotoxicity Targets for MK

The drug target prediction workflow was conducted as follows: First, MK was searched in the PubChem database, and its name and molecular formula were verified before downloading the SDF file of its 2D structure. Subsequently, this file was transferred up the SwissTarget Prediction database (http://www.swisstargetprediction.ch/) (accessed on 15 September 2025) for target prediction, and the predicted potential targets of MK were exported. Disease-related targets were obtained through the GeneCards database (http://www.genecards.org/) (accessed on 15 September 2025) using “neurotoxicity” as the search keyword to collect associated disease targets. Finally, the obtained potential targets of MK and neurotoxicity-related disease targets were separately uploaded to the Venn online tool (http://bioinformatics.psb.ugent.be/webtools/Venn/) (accessed on 18 September 2025) to generate a Venn diagram, through which the intersecting targets—identified as key targets through which MK may exert neurotoxic effects—were determined.

### 2.4. Construction of the Protein–Protein Interaction (PPI) Network and KEGG Pathway Analysis Were Performed

The PPI network was constructed primarily using the STRING database (https://cn.string-db.org/) (accessed on 18 September 2025). The overlapping targets obtained from the previously generated Venn diagram of MK and neurotoxicity were uploaded to the STRING database, with the species specified as Homo sapiens, to acquire the PPI network diagram. Cytoscape v3.10.3 software was utilized for the analysis and optimization of the network structure and key nodes. Detailed results are provided in Table S1. To elucidate the biological processes and pathways involved in MK-induced neurotoxicity, KEGG pathway analysis was performed using the DAVID database (https://davidbioinformatics.nih.gov/summary.jsp) (accessed on 14 October 2025). The intersecting targets from the Venn diagram were uploaded to the database, with the species set to human, and the results were subsequently retrieved. From the obtained results, the top 20 entries in the KEGG category were selected and ranked in ascending order of *p*-value.

### 2.5. MK Exposure and General Developmental Toxicity Assessment

The median lethal concentration (LC_50_) of MK on zebrafish embryos was first determined through an acute exposure experiment to assess its toxicity. Embryos were inoculated into 6-well plates at a density of 10 per well and exposed to MK solutions with concentrations of 50, 100, 200, 400, 800, and 1600 μg/L, respectively. The detailed/specific results are presented in Table S3. The exposure solution was replaced daily, and observations were conducted 3 times daily, continuing up to 96 h post-fertilization (hpf). Two control groups were established simultaneously: (1) Blank control group: using aerated water meeting standard specifications for all parameters; (2) Solvent control group (DMSO): adding the same volume of DMSO as in the highest concentration drug group (0.0002% (*v*/*v*)). The experiment was performed with three replicates, and the LC_50_ at 96 hpf was calculated to be 0.2 mg/L. Based on these results, four exposure concentrations—0 (control), 0.2, 2, and 20 μg/L—were established for the formal experiment. The exposure system for each concentration group consisted of ten embryos placed in wells containing 5 mL of aerated water, with each concentration tested in three independent replicates. At 24, 72, and 144 hpf, developmental parameters including mortality, hatching rate, and malformation rate were systematically recorded using a fluorescence stereomicroscope system. The following parameters were assessed at designated time points: yolk sac area and spontaneous movement (24 hpf), heart rate (72 hpf), swim bladder area (144 hpf), and body length (72 and 144 hpf) (*n* = 15). The concentrations used in this exposure experiment (0.2, 2, and 20 µg/L) were set based on the 96-h acute LC_50_ (200 µg/L). The highest exposure concentration was designated as one-tenth of the acute LC_50_ (20 µg/L) to adhere to the precautionary principle in ecotoxicology and ensure the observation of sublethal effects during chronic exposure. This concentration range also exhibits greater environmental relevance.

### 2.6. Locomotor Behavior Testing in Zebrafish Larvae

Following exposure to MK until 144 hpf, morphologically normal larvae were randomly chosen from each concentration group and individually transferred to 24-well plates containing 2 mL of aerated water for neurobehavioral assessment. After a 10-min acclimation period in the DanioVision tracking system, their movement trajectories were recorded under 28 ± 0.5 °C conditions during a 50-min light-dark cycle stimulation (10 min light/10 min dark). Three independent experimental replicates were performed for each concentration group. Behavioral parameters including locomotor distance, velocity, acceleration, and cumulative movement duration were subsequently analyzed using EthoVision XT 16.0 software. Specifically, to assess neurobehavioral abnormalities, two derived behavioral metrics were defined: (1) Freezing time: the cumulative duration during the 50-min test period when larvae exhibited sustained instantaneous velocity ≤ 0.1 cm/s; (2) Frenetic/manic state: active behavior characterized by instantaneous velocity ≥ 2.0 cm/s with acceleration ≥ 0.1 cm/s^2^, quantified by both “total duration of frenetic state” and “frequency of frenetic episodes.”.

### 2.7. Investigation of Neurodevelopmental Toxicity in Zebrafish Larvae

To evaluate the effects of MK on neural development in zebrafish, embryos of transgenic strains *Tg(huc:eGFP)* and *Tg(hb9:eGFP)* were exposed to varying concentrations of MK solutions. For each concentration, three replicates were established, with each replicate consisting of 30 embryos placed in Petri dishes containing 20 mL of exposure solution. Microscopic observation was performed at specific time points. At 72 hpf and 144 hpf, 15 larvae were randomly selected from each treatment group and fixed with 4% paraformaldehyde for 2 h. Subsequently, images were acquired using a fluorescence stereomicroscope (Nikon SMZ25, Tokyo, Japan) (Figure S2), and the fluorescence intensity of the *Tg(huc:eGFP)* strain as well as the axonal length of the *Tg(hb9:eGFP)* strain were statistically analyzed. All quantitative analyses of neurodevelopmental indicators were performed using NIS-Elements D 5.20.00 software.

### 2.8. Transcriptome Analysis and Quantitative Real-Time PCR

To investigate the potential toxicological pathways of MK, total RNA was extracted from 100 zebrafish larvae in the 0 μg/L and 0.2 μg/L treatment groups. Volcano plots were used to visualize genes that are differentially transcribed, and Kyoto Encyclopedia of Genes and Genomes (KEGG) enrichment analysis was conducted to evaluate the distribution characteristics and functional roles of these genes. Following this analysis, total RNA was extracted from zebrafish exposure groups (0, 0.2, 2, and 20 μg/L) using TRIzol reagent, with three replicates per group. Following cDNA synthesis with the PrimeScript^®^ RT reagent kit, gene expression levels were analyzed on a Bio-Rad CFX Connect Real-Time PCR System (Shanghai, China) employing the SYBR Green detection method. The expression levels of neurodevelopment-related genes (*elavl3*, *mbp*, *nrd*, *gap43*), oxidative stress-related genes (*nrf2*, *cat*, *cu/zn-sod*, *mn-sod*), calcium signaling pathway-related genes (*kdr*, *atp2a1*, *cacnalab*, *ppp3ca*), and apoptosis-related genes (*caspase 9*, *bax*, *p53*, *caspase3*) were quantified using primers synthesized by Sangon Biotech (Shanghai, China), as detailed in Table S2.

### 2.9. Data Analytics

To prevent bias, the researchers administering the treatments were blinded throughout the study. We performed all statistical analyses with SPSS 18.0. We tested all data for homogeneity of variance. For data with homogeneous variance, we used the Student-Newman-Keuls (SNK) test for post hoc pairwise comparisons following a significant one-way ANOVA. For data with non-homogeneous variance, we used Tamhane’s T2 test for post hoc comparisons. Data are expressed as mean ± SEM, and a *p*-value < 0.05 was defined as statistically significant.

## 3. Results

### 3.1. Network Toxicological Analysis of MK Neurotoxicity

**Figure 1:**
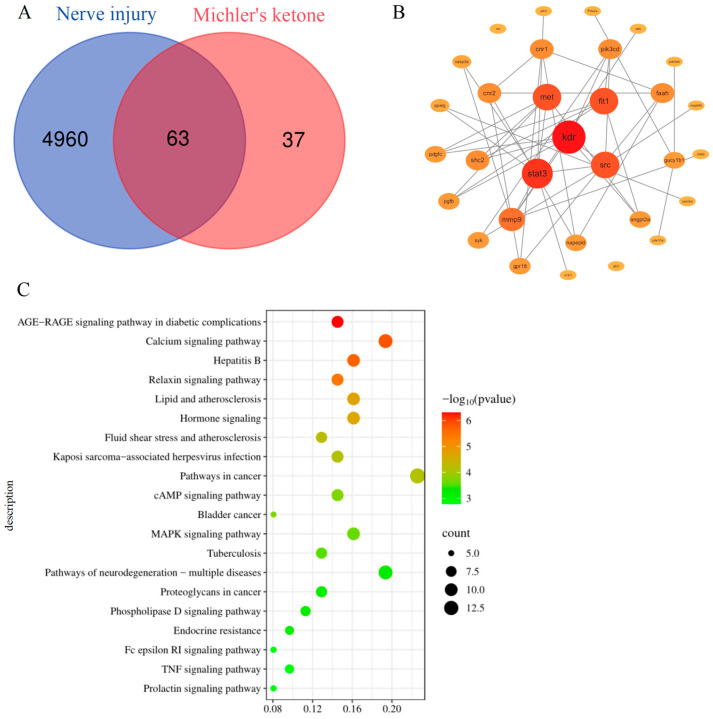
Network toxicology-based prediction of MK toxicity. (**A**) Venn diagram analysis of shared targets in MK-induced toxicity and neurological injury. (**B**) Construction of a protein Interaction network for core potential targets. The larger the degree, the darker the color. (**C**) Enrichment analysis of potential targets in KEGG pathways.

In the current investigation, 100 potential targets of MK and 5023 neurotoxicity-related targets were identified using the SwissTargetPrediction and GeneCards databases, respectively. Following integration, removal of duplicates, and Venn analysis, 63 intersecting targets were obtained and defined as potential targets for MK-induced neurotoxicity (Figure 1A). A PPI network was constructed using the STRING database, and topological analysis was subsequently performed with Cytoscape software to generate a visualized PPI network (Figure 1B). The core target *kdr* was identified through this process. The network not only reveals complex interactions among the potential targets but also intuitively reflects the degree centrality of each node through variations in node size and color gradient.

Further KEGG pathway enrichment analysis was performed on the aforementioned 63 potential targets using the DAVID database to identify the major signaling pathways involved. A bubble plot was generated displaying the top 20 significantly enriched pathways ranked in ascending order based on *p*-values (Figure 1C), primarily including: AGE-RAGE signaling pathway in diabetic complications, Calcium signaling pathway, Hepatitis B, and Relaxin signaling pathway.

### 3.2. Effects of MK Exposure on the Early Development of Zebrafish Larvae

**Figure 2:**
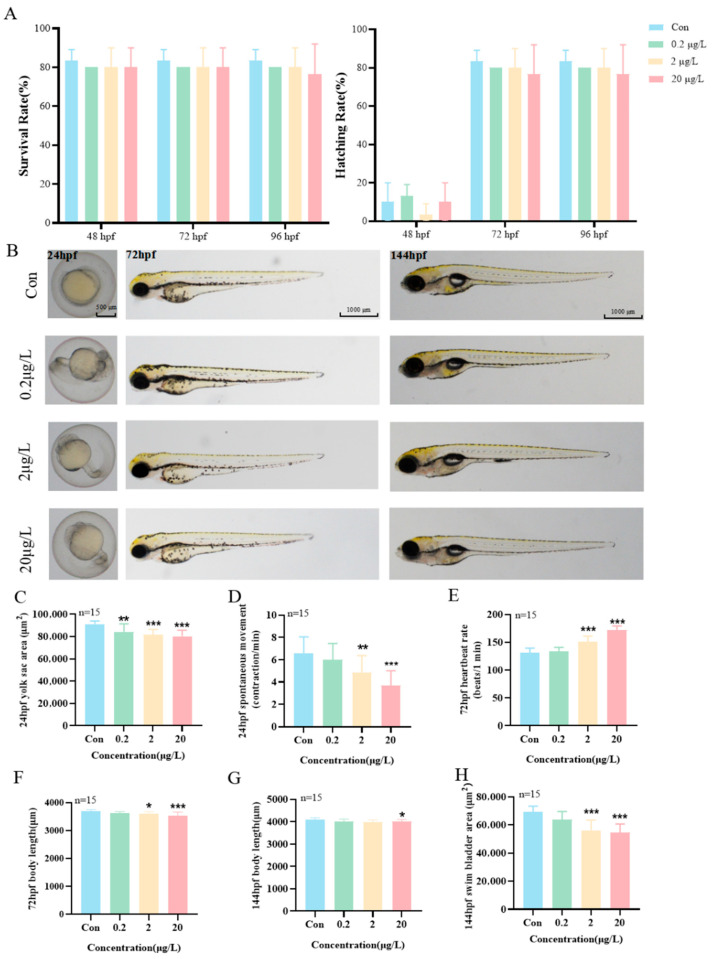
Effects of MK exposure on the early development of zebrafish larvae. (**A**) Survival rate and Hatching rate exposed until 96 hpf. (**B**) Representative images of early developmental stages. (**C**) Yolk sac area and (**D**) number of spontaneous movements exposed until 24 hpf. (**E**) Heart rate and (**F**) body length exposed until 72 hpf. (**G**) Body length and (**H**) swim bladder area exposed until 144 hpf. *p*-values: *p* < 0.001 (***), *p* < 0.01 (**), and *p* < 0.05 (*).

Adverse effects on early growth and development were observed in zebrafish larvae following exposure to MK. Zebrafish embryos at 2 hpf were exposed to varying concentrations of MK (0.2, 2, and 20 μg/L), with developmental phenotypes continuously monitored. The results demonstrated that no significant differences in survival or hatching rates were observed in zebrafish embryos exposed to MK concentrations ≤ 20 μg/L compared to the control group (Figure 2A). At 24 hpf, a significant reduction in yolk sac area was observed in the 0.2 μg/L MK exposure group compared to the control (Figure 2C, *p* < 0.01), while the number of spontaneous movements per minute was significantly decreased in embryos exposed to 2 μg/L and higher concentrations (Figure 2D, *p* < 0.01). By 72 hpf, significantly increased heart rates were recorded in zebrafish larvae exposed to 2 μg/L and higher MK concentrations (Figure 2E, *p* < 0.001), accompanied by inhibited body length growth with a 2.2% reduction (Figure 2F, *p* < 0.05)-Although this body length reduction may seem modest, it occurs during a critical window of early zebrafish development and co-occurs with heart rate abnormalities and subsequent neurodevelopmental impairments. This constellation of effects is considered to reflect MK’s systemic disruption of development and demonstrates clear biological relevance. When exposed until 144 hpf, significantly suppressed body length was observed in the 20 μg/L MK treatment group, showing a 1.9% reduction (Figure 2G, *p* < 0.05), along with markedly decreased swim bladder area (Figure 2H, *p* < 0.001). These combined effects collectively exacerbated the locomotor behavioral abnormalities in the larvae, thereby confirming their biological relevance.

### 3.3. Effects of MK on Locomotor Behavior in Zebrafish Larvae

To investigate the effects of MK on locomotor behavior in zebrafish larvae at environmentally relevant concentrations, embryos were exposed until 144 hpf, and their behavioral changes were assessed under light-dark alternating stimulation conditions.

**Figure 3:**
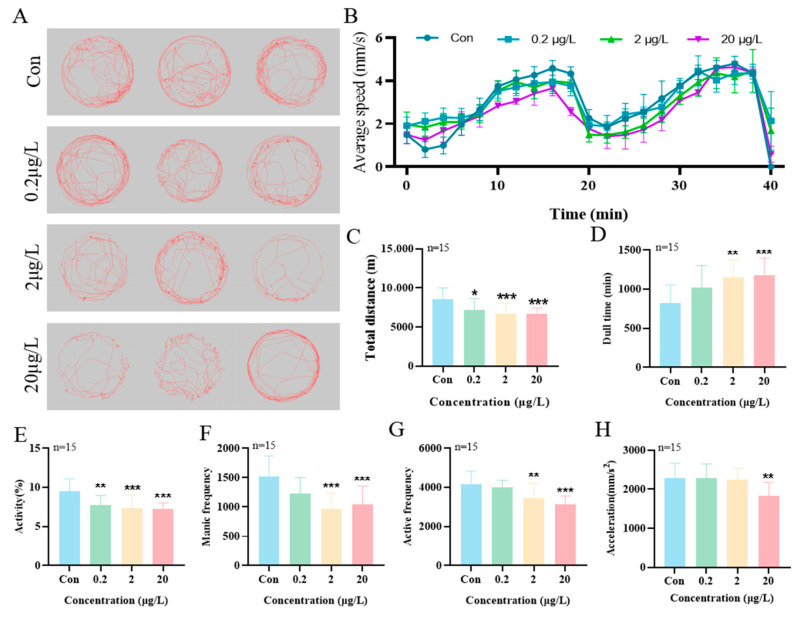
Effects of MK exposure on locomotor behavior in zebrafish larvae. (**A**) Locomotor tracks, (**B**) average velocity, (**C**) total distance traveled, (**D**) freezing time, (**E**) activity level, (**F**) manic-like episodes, (**G**) active frequency, and (**H**) acceleration in zebrafish larvae exposed until 144 hpf. *p*-values: *p* < 0.001 (***), *p* < 0.01 (**), and *p* < 0.05 (*).

Figure 3A displays the movement trajectories and heat maps of zebrafish larvae from different MK exposure groups. Under light-dark stimulation, a concentration-dependent decrease in larval movement velocity was observed with increasing MK concentrations (Figure 3B). The total movement distance was reduced by 16.0% compared to the control group (Figure 3C), with significant differences observed starting from 0.2 μg/L (*p* < 0.05). Concurrently, a significant prolongation of freezing time by approximately 40.6% was recorded (Figure 3D, *p* < 0.01). Furthermore, MK exposure was found to differentially affect larval activity levels, frenzy states, and active frequency (Figure 3E–G). At the 20 μg/L concentration, larval acceleration was significantly suppressed (Figure 3H, *p* < 0.001). These results collectively demonstrate that MK exposure exerts a significant inhibitory effect on the locomotor behavior of zebrafish larvae.

### 3.4. Effects of MK on the Nervous System of Zebrafish Larvae

**Figure 4:**
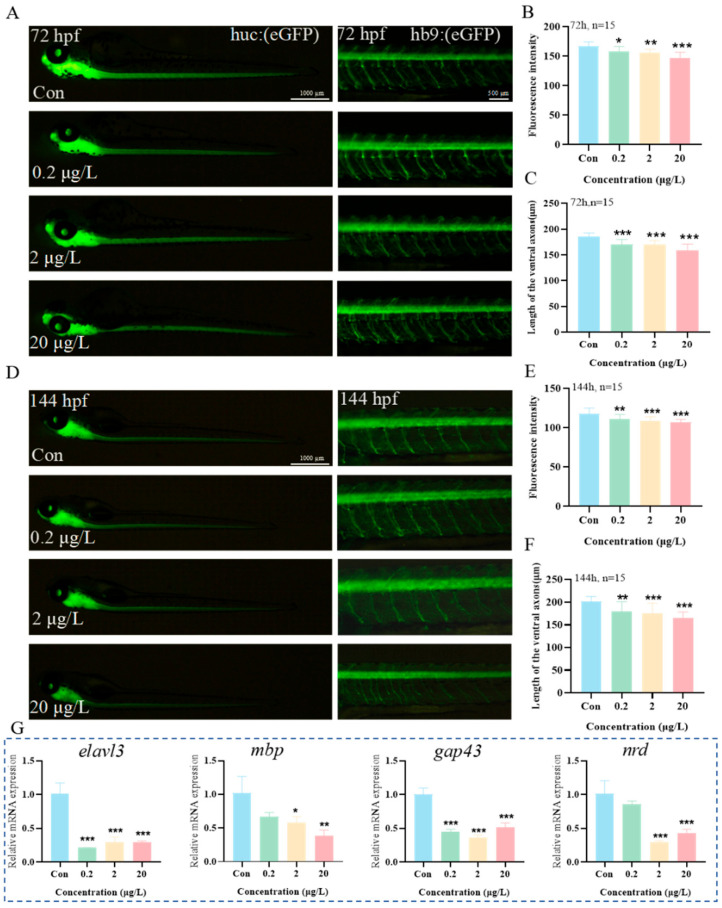
Effects of MK exposure on neural development in zebrafish larvae. Representative images of transgenic zebrafish lines (**A**) *Tg(huc:eGFP)* and *Tg(hb9:eGFP)* exposed until 72 hpf. (**B**) Fluorescence intensity and (**C**) axon length in zebrafish larvae at 72 hpf. Representative images of transgenic zebrafish lines (**D**) *Tg(huc:eGFP)* and *Tg(hb9:eGFP)* exposed until 144 hpf. (**E**) Fluorescence intensity and (**F**) axon length in zebrafish larvae at 144 hpf. (**G**) Neural development-related gene expression. *p*-values: *p* < 0.001 (***), *p* < 0.01 (**), and *p* < 0.05 (*).

The effects of MK on neural development in zebrafish were further evaluated using transgenic zebrafish lines: the central nervous system marker strain *Tg(huc:eGFP)* and the motor neuron marker strain *Tg(hb9:eGFP)*. Larvae were exposed until 72 hpf and 144 hpf, followed by examination using fluorescence stereomicroscopy combined with quantitative image analysis (Figure 4). The results demonstrated that at 72 hpf, with increasing MK concentrations, a reduction in green fluorescence intensity in the brain and spinal cord regions of *Tg(huc:eGFP)* zebrafish, as well as a decrease in motor neuron axon length in *Tg(hb9:eGFP)* zebrafish, were observed starting from 0.2 μg/L, showing reductions of 4.9% and 8.6%, respectively (Figure 4A–C). By 144 hpf, compared to the control group, exposure to 0.2 μg/L MK continued to significantly suppress fluorescence intensity in the brain and spinal cord and motor neuron axon length, with reductions of 5.8% and 10.8%, respectively (Figure 4D–F, *p* < 0.01). Additionally, the expression levels of neurodevelopment-related genes (*elavl3*, *mbp*, *nrd*, *gap43*) were analyzed by qPCR, and MK was found to suppress the expression of these genes (Figure 4G). These results collectively indicate that MK exerts significant damaging effects on the nervous system development of zebrafish larvae.

### 3.5. MK Induces Oxidative Stress to Disrupt Calcium Ion Signaling Pathway

**Figure 5:**
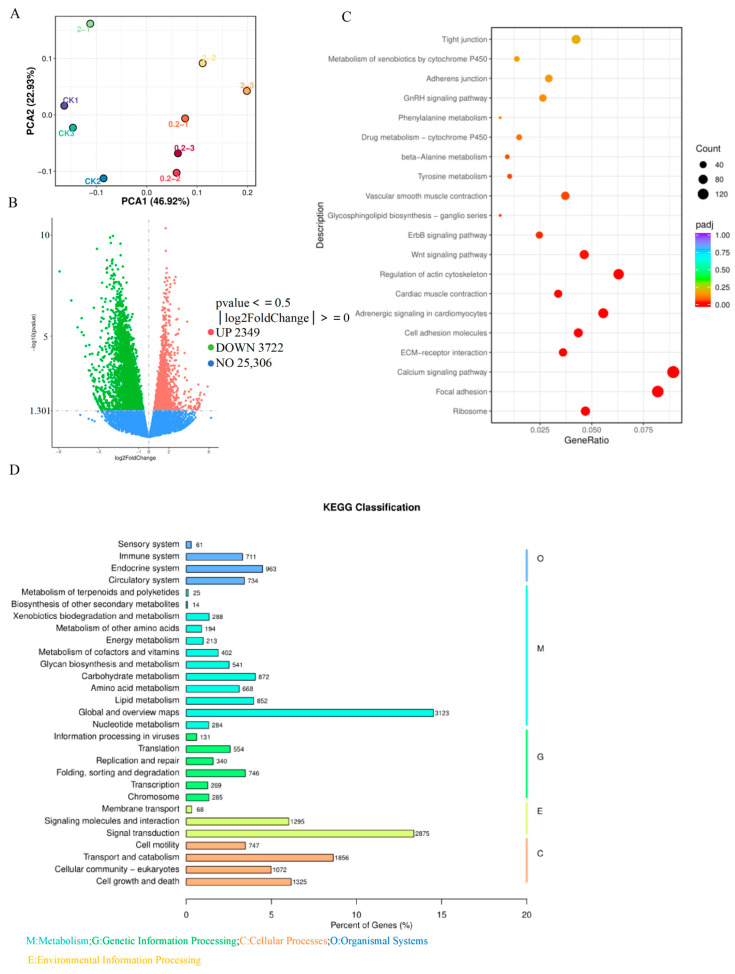
Transcriptomic profiling of zebrafish larvae in response to MK exposure. (**A**) PCA scatter plot comparing the exposure group versus the control group. (**B**) Volcano plot of differentially DEGs screened from the 0.2 μg/L exposure group. (**C**) GO functional enrichment analysis of DEGs identified under 0.2 μg/L exposure. (**D**) KEGG pathway annotation statistics for the 0.2 μg/L exposure group.

**Figure 6:**
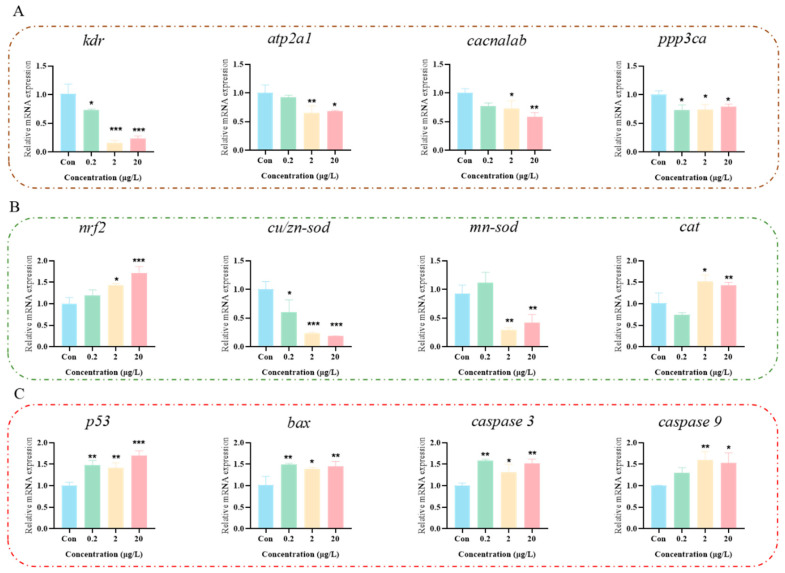
Gene expression in zebrafish larvae. (**A**) Calcium signaling pathway-related genes. (**B**) Oxidative stress-related genes. (**C**) Apoptosis-related genes. *p*-values: *p* < 0.001 (***), *p* < 0.01 (**), and *p* < 0.05 (*).

To further investigate the neurotoxic mechanism of MK in zebrafish larvae, a transcriptomic analysis was performed using the control group and the 0.2 μg/L MK-exposed group. Principal component analysis (PCA) showed clear intergroup separation with minimal intragroup variation (Figure 5A). Compared to the control group, 2349 genes were found to be up-regulated and 3722 genes were down-regulated in the MK-exposed group (Figure 5B). The KEGG enrichment scatter plot revealed that pathways such as Ribosome, Focal adhesion, and Calcium signaling pathway were significantly enriched (Figure 5C). The KEGG classification is displayed in Figure 5D, which was mainly categorized under Global and overview maps and Signal transduction. Based on the integration of network toxicology and transcriptomic results, the calcium signaling pathway was suggested to play a critical role in MK-induced neurotoxicity. To validate the effect of MK on the calcium signaling pathway, the expression levels of related genes (*kdr*, *atp2a1*, *cacnalab*, *ppp3ca*) were examined (Figure 6A). It was observed that the expression of *kdr*, *atp2a1*, and *cacnala* was reduced at an MK concentration of 2 μg/L, while the expression of ppp3ca was significantly decreased even at 0.2 μg/L. In addition, altered expression was detected in oxidative stress-related genes (*nrf2*, *cat*, *cu/zn-sod*, *mn-sod*) (Figure 6B) and apoptosis-related genes (*p53*, *caspase 3*, *caspase 9*, *bax*) (Figure 6C), suggesting that oxidative stress was induced by MK in zebrafish, subsequently leading to apoptosis. In summary, this study demonstrates that MK induces neurodevelopmental damage and locomotor abnormalities in zebrafish larvae, which may be mediated through the induction of oxidative stress, disruption of calcium signaling, and subsequent triggering of apoptosis.

## 4. Discussion

This study integrated network toxicology predictions, in vivo phenotypic observations in zebrafish, transcriptomic pathway enrichment analysis, and qPCR targeted validation to establish a complete evidence chain spanning from target prediction and pathway screening to toxicity effect confirmation. This approach systematically investigated the molecular mechanism of MK-induced developmental neurotoxicity. By correlating molecular responses with phenotypic toxicity, the study not only confirmed the neurotoxic effects of MK but also preliminarily revealed its underlying mechanism of action.

Zebrafish have been established as an ideal model for neurotoxicity assessment due to their transparent embryos, high genetic homology with humans (approximately 70%), partially conserved brain structure, and capacity for automated behavioral tracking. Transgenic fluorescence technology further enables direct visualization of neural network function [10]. In this study, MK exposure was observed to result in reduced yolk sac area and decreased spontaneous movements in zebrafish embryos at 24 hpf, suggesting early impairment of the nervous system and motor pathways [11]. Significant body length shortening was demonstrated at both 72 hpf and 144 hpf, a phenotype that aligns with effects caused by known neurodevelopmental toxicants [12], thereby providing additional support for the inference that MK interferes with neural development. Locomotor behavior serves as a critical indicator for evaluating neurological function. In the present investigation, zebrafish larvae exposed to MK until 144 hpf were observed to exhibit reduced total movement distance and prolonged freezing time, indicating significant suppression of motor capability. This behavioral phenotype corresponds with neurotoxic effects induced by various environmental contaminants [13].

Locomotor deficits are closely associated with impaired neurogenesis. To further validate the impact of MK exposure on the nervous system of zebrafish larvae, transgenic zebrafish lines *Tg(huc:eGFP)* and *Tg(hb9:eGFP)* were employed for neural morphological assessment. The huc gene (i.e., elavl3 gene) is a specific marker for early neurons in zebrafish. By inserting the green fluorescent protein (*GFP*) gene into the promoter sequence of huc, the huc-GFP transgenic zebrafish line was generated. In this strain of zebrafish, the neurons in the central nervous system (including the brain and spinal cord) can be clearly localized and stably exhibit green fluorescence. The hb9-*GFP* transgenic zebrafish was constructed based on the hb9 gene, which is a key regulatory gene for motor neuron development in zebrafish. Its promoter drives specific *GFP* expression in motor neurons [14]. This zebrafish line is primarily used for observing axonal length of motor neurons. The results demonstrated that MK exposure induced reduced fluorescence intensity in the spinal cord and shortened axonal length at both 72 hpf and 144 hpf, indicating impaired neuronal development and abnormal motor neuron structure. Similar studies have revealed that structural damage to motor neurons can lead to signal transmission deficits, subsequently resulting in decreased swimming capacity [15]. At the molecular level, MK exposure led to the downregulation of several key neurodevelopmental genes (*elavl3*, *mbp*, *nrd*, *gap43*). Among these, *mbp* encodes myelin basic protein, and its reduced expression can cause deterioration of myelin structure and axonal atrophy [16]. The genes *nrd* and *gap43* are involved in the regulation of neuronal differentiation and axonal growth, respectively [17,18,19]. The suppression of these genes further confirms that MK interferes with the normal development of the nervous system in zebrafish.

ROS accumulation-induced oxidative stress is recognized as a significant trigger for various neurological disorders [20] and has been identified as a key common event in developmental neurotoxicity [21]. Within the cellular antioxidant defense system, *Nrf2* serves as the central transcriptional regulator [22]. Superoxide dismutase (SOD) is one of the key detoxification agents in the human body, which effectively clears superoxide anions and converts them into hydrogen peroxide (H_2_O_2_) [23]. Catalase (CAT), primarily localized in peroxisomes, effectively catalyzes the decomposition of H_2_O_2_ into water and oxygen [24]. When superoxide accumulation occurs in vivo, it is often accompanied by enhanced CAT activity to counteract oxidative stress. The results of this study demonstrate that MK exposure induces abnormal expression of oxidative stress-related genes in zebrafish larvae, confirming its capacity to trigger oxidative stress in the organism. Furthermore, integrated network toxicology and transcriptomic KEGG enrichment analysis revealed that the calcium signaling pathway plays a significant role in MK-induced neurotoxicity. Previous studies have established that calcium overload promotes ROS generation [25], while increased ROS levels further exacerbate intracellular Ca^2+^ elevation, forming a self-amplifying positive feedback loop [26]. In this study, disrupted expression of calcium signaling pathway-related genes (*kdr*, *atp2a1*, *cacnalab*, *ppp3ca*) was observed in zebrafish larvae, corroborating the aforementioned mechanism. Regarding the apoptosis pathway, *p53* functions as both a central regulator of the cell cycle and a key regulatory protein in genotoxic stress-induced apoptosis [27], capable of activating the pro-apoptotic gene *bax* [28]. *Caspase-9* serves as the core initiator of the endogenous mitochondrial apoptosis pathway [29], while *Caspase-3* acts as the key executioner connecting apoptosis and pyroptosis processes [30]. The observed aberrant expression of these apoptosis-related genes in this study further supports that MK can trigger the apoptotic process. This research indicates that sustained oxidative stress can induce dysregulation of calcium signaling in endothelial cells, leading to calcium overload. Calcium overload significantly suppresses *kdr* gene expression, impairs blood–brain barrier repair capacity, and becomes a critical factor in inducing endothelial cell apoptosis. Research has demonstrated that *kdr* is confirmed as one of the core targets in pyrethroid (PYs)-induced neurotoxicity studies, participating in the process of neural injury through a vascular-neural interaction regulatory mechanism [31]. We recognize that this study did not directly assess blood–brain barrier (BBB) metrics, and therefore cannot precisely quantify the extent of BBB impairment attributable to *kdr* downregulation. We explicitly identify this as an important direction for future research. The value of the present work lies in integrating the “KDR-BBB-neural injury” pathway—which aligns with established scientific consensus—into the mechanistic framework, thereby providing a more comprehensive explanatory model for MK-induced neurotoxicity. Validating this pathway will be a primary focus of subsequent work aimed at fully elucidating the underlying vascular-neural interplay. In summary, this study demonstrates that MK induces neurodevelopmental damage and locomotor abnormalities in zebrafish larvae, which may be mediated through the induction of oxidative stress, disruption of calcium signaling, and subsequent triggering of apoptosis.

## 5. Conclusions

In summary, the neurotoxic mechanism of MK was systematically investigated in this study using zebrafish as a model, integrating network toxicology prediction, transcriptomic analysis, and RT-qPCR validation. It was confirmed that MK exposure could induce oxidative stress in zebrafish larvae, which further disrupted the calcium ion signaling pathway and triggered cell apoptosis, ultimately leading to neurodevelopmental and motor behavioral disorders. A complete evidence chain from effect prediction to mechanism verification was established in this study. It not only provided key data for the scientific assessment of the aquatic ecological risks of MK but also offered a new theoretical perspective for analyzing the molecular mechanisms by which pollutants interfere with neurodevelopment, thus exerting positive significance for the research and development in related fields.

Future studies could focus on the following aspects: (1) While this study confirmed *kdr* as a core target and identified an interaction between the calcium signaling pathway and oxidative stress, the specific molecular mechanisms require further elucidation through techniques such as in vitro cellular assays, molecular docking, and protein–protein interaction validation. (2) Evaluation of the combined neurotoxicity of MK with other environmental co-pollutants to more accurately reflect real-world environmental risks. (3) Given the cumulative nature of MK in the environment, future research should investigate its transgenerational neurotoxicity in adult fish and their offspring, extending beyond the larval stage focused on in this study. These investigations will further refine the theoretical framework of aromatic ketone-induced neurotoxicity and provide more comprehensive support for ecological security and public health protection.

## Data Availability

The raw data supporting the conclusions of this article will be made available by the authors upon request.

## Supplementary Materials

The following supporting information can be downloaded at: https://www.mdpi.com/article/10.3390/biology15010003/s1. Table S1: Candidate targets screened from the PPI network. Table S2: The primer sequences for QPCR. Table S3: Acute toxicity results from exposure to MK. Figure S1: Guidelines for the care and use of laboratory animals. Figure S2: Region for fluorescence intensity quantification (*Tg(huc:eGFP)* embryo).

## References

[1] Krishnamoorthy S., Allabasha N., Mani M.K., Sarkar A.K. (2022). Michler’s Ketone: Beta-Cyclodextrin Host-Guest Inclusion Complex for Enhancing the Ultraviolet Protection Factor of Poplin Cotton Fabric. Photochem. Photobiol..

[2] Ozaki A., Kawasaki C., Kawamura Y., Tanamoto K. (2006). Migration of bisphenol A and benzophenones from paper and paperboard products used in contact with food. Shokuhin Eiseigaku Zasshi.

[3] Ozaki A., Yamaguchi Y., Fujita T., Kuroda K., Endo G. (2004). Chemical analysis and genotoxicological safety assessment of paper and paperboard used for food packaging. Food Chem. Toxicol..

[4] Parodi S., Santi L., Russo P., Albini A., Vecchio D., Pala M., Ottaggio L., Carbone A. (1982). DNA damage induced by auramine O in liver, kidney, and bone marrow of rats and mice, and in a human cell line (alkaline elution assay and SCE induction). J. Toxicol. Environ. Health.

[5] Williams G.M., Laspia M.F., Dunkel V.C. (1982). Reliability of the hepatocyte primary culture/DNA repair test in testing of coded carcinogens and noncarcinogens. Mutat. Res..

[6] Lafi A., Parry J.M., Parry E.M. (1986). The effect of Michler’s ketone on cell division, chromosome number and structure in cultured Chinese hamster cells. Mutagenesis.

[7] Han X., Nabb D.L., Mingoia R.T., Yang C.H. (2007). Determination of xenobiotic intrinsic clearance in freshly isolated hepatocytes from rainbow trout (*Oncorhynchus mykiss*) and rat and its application in bioaccumulation assessment. Environ. Sci. Technol..

[8] Gibert Y., Trengove M.C., Ward A.C. (2013). Zebrafish as a genetic model in pre-clinical drug testing and screening. Curr. Med. Chem..

[9] Planchart A., Mattingly C.J., Allen D., Ceger P., Casey W., Hinton D., Kanungo J., Kullman S.W., Tal T., Bondesson M. (2016). Advancing toxicology research using in vivo high throughput toxicology with small fish models. ALTEX.

[10] Hughes S., Hessel E.V.S. (2024). Zebrafish and nematodes as whole organism models to measure developmental neurotoxicity. Crit. Rev. Toxicol..

[11] Ravenscroft G., Sollis E., Charles A.K., North K.N., Baynam G., Laing N.G. (2011). Fetal akinesia: Review of the genetics of the neuromuscular causes. J. Med. Genet..

[12] Zhang L., Li X., Yuan Q., Sun S., Liu F., Liao X., Lu H., Chen J., Cao Z. (2024). Isavuconazole Induces Neurodevelopment Defects and Motor Behaviour Impairment in Zebrafish Larvae. Mol. Neurobiol..

[13] Zhang Q., Zheng S., Shi X., Luo C., Huang W., Lin H., Peng J., Tan W., Wu K. (2023). Neurodevelopmental toxicity of organophosphate flame retardant triphenyl phosphate (TPhP) on zebrafish (*Danio rerio*) at different life stages. Environ. Int..

[14] Gu J., Guo M., Yin X., Huang C., Qian L., Zhou L., Wang Z., Wang L., Shi L., Ji G. (2022). A systematic comparison of neurotoxicity of bisphenol A and its derivatives in zebrafish. Sci. Total Environ..

[15] Wang X., Hu M., Li M., Huan F., Gao R., Wang J. (2024). Effects of exposure to 3,6-DBCZ on neurotoxicity and AhR pathway during early life stages of zebrafish (*Danio rerio*). Ecotoxicol. Environ. Saf..

[16] Verdu E., Ceballos D., Vilches J.J., Navarro X. (2000). Influence of aging on peripheral nerve function and regeneration. J. Peripher. Nerv. Syst..

[17] Mueller T., Wullimann M.F. (2002). Expression domains of neuroD (nrd) in the early postembryonic zebrafish brain. Brain Res. Bull..

[18] Kowara R., Menard M., Brown L., Chakravarthy B. (2007). Co-localization and interaction of DPYSL3 and GAP43 in primary cortical neurons. Biochem. Biophys. Res. Commun..

[19] Frey D., Laux T., Xu L., Schneider C., Caroni P. (2000). Shared and unique roles of CAP23 and GAP43 in actin regulation, neurite outgrowth, and anatomical plasticity. J. Cell Biol..

[20] Farkhondeh T., Mehrpour O., Forouzanfar F., Roshanravan B., Samarghandian S. (2020). Oxidative stress and mitochondrial dysfunction in organophosphate pesticide-induced neurotoxicity and its amelioration: A review. Environ. Sci. Pollut. Res. Int..

[21] Nishimura Y., Kanda Y., Sone H., Aoyama H. (2021). Oxidative Stress as a Common Key Event in Developmental Neurotoxicity. Oxidative Med. Cell. Longev..

[22] Lal R., Dharavath R.N., Chopra K. (2024). Nrf2 Signaling Pathway: A Potential Therapeutic Target in Combating Oxidative Stress and Neurotoxicity in Chemotherapy-Induced Cognitive Impairment. Mol. Neurobiol..

[23] Rodriguez-Fuentes G., Rubio-Escalante F.J., Norena-Barroso E., Escalante-Herrera K.S., Schlenk D. (2015). Impacts of oxidative stress on acetylcholinesterase transcription, and activity in embryos of zebrafish (*Danio rerio*) following Chlorpyrifos exposure. Comp. Biochem. Physiol. C Toxicol. Pharmacol..

[24] Li X., Zhou S., Qian Y., Xu Z., Yu Y., Xu Y., He Y., Zhang Y. (2018). The assessment of the eco-toxicological effect of gabapentin on early development of zebrafish and its antioxidant system. RSC Adv..

[25] Brookes P.S., Yoon Y., Robotham J.L., Anders M.W., Sheu S.S. (2004). Calcium, ATP, and ROS: A mitochondrial love-hate triangle. Am. J. Physiol. Cell Physiol..

[26] Peng T.I., Jou M.J. (2010). Oxidative stress caused by mitochondrial calcium overload. Ann. N. Y. Acad. Sci..

[27] Weber K.J., Wenz F. (2002). p53, apoptosis and radiosensitivity–experimental and clinical data. Onkologie.

[28] Hao Q., Chen J., Lu H., Zhou X. (2023). The ARTS of p53-dependent mitochondrial apoptosis. J. Mol. Cell Biol..

[29] Johnson C.R., Jarvis W.D. (2004). Caspase-9 regulation: An update. Apoptosis.

[30] Jiang M., Qi L., Li L., Li Y. (2020). The caspase-3/GSDME signal pathway as a switch between apoptosis and pyroptosis in cancer. Cell Death Discov..

[31] Lin Y., Zeng W., Zhang Y., Hu Y., Liang Z., Chen Z., Lu Z., Zhang W., Wang Z., Jiang Y. (2025). Neurotoxicity mechanisms of pyrethroids studied by network toxicology and molecular docking. Toxicol. Mech. Methods.

