# Supplementary material for: Investigation of the Neurotoxic Effects and Mechanisms of Michler’s Ketone as Investigated by Network Toxicology and Transcriptomics
Source: Biology (Basel). 2025 Dec 19;15(1):3. doi: 10.3390/biology15010003 (PMC12785016; doi:10.3390/biology15010003)

Supplementary Materials

Supplementary Table S1

Candidate targets screened from the PPI network.

Name	Degree	Closeness centrality	Betweenness centrality	Topological coefficient
kdr	8	0.5365853658536586	0.22877122877122874	0.2857142857142857
stat3	7	0.55	0.4063436563436563	0.1836734693877551
src	6	0.5365853658536586	0.20917574489003055	0.28431372549019607
met	6	0.5116279069767442	0.1729698872556015	0.3076923076923077
flt1	6	0.39285714285714285	0.03954378954378954	0.4375
mmp9	5	0.4888888888888889	0.20979020979020976	0.24615384615384617
pik3cd	4	0.4313725490196078	0.022156415013557865	0.5
shc2	4	0.44897959183673475	0.009041751898894754	0.5
faah	4	1.0	0.05555555555555555	0.875
cnr2	4	1.0	0.05555555555555555	0.875
cnr1	4	1.0	0.05555555555555555	0.875
syk	3	0.4	0.0035797535797535795	0.5454545454545454
pgfb	3	0.4150943396226415	0.009041751898894754	0.7083333333333334
pdgfc	3	0.4150943396226415	0.009041751898894754	0.7083333333333334
gpr18	3	0.8	0.0	1.0
napepld	3	0.8	0.0	1.0
angpt2a	3	0.4150943396226415	0.009041751898894754	0.7083333333333334
gucy1b1	3	1.0	1.0	0.0
pparg	2	0.3728813559322034	0.09090909090909091	0.5
casp3a	2	0.3859649122807018	0.0	0.6
pparaa	1	0.275	0.0	0.0
nr3c1	1	0.360655737704918	0.0	0.0
mapk8b	1	0.3548387096774193	0.0	0.0
jak3	1	0.360655737704918	0.0	0.0
pde5ab	1	0.6	0.0	0.0
pde10a	1	0.6	0.0	0.0
ret	1	0.3384615384615385	0.0	0.0
ctsk	1	0.3333333333333333	0.0	0.0
ctsba	1	0.3333333333333333	0.0	0.0
abl1	1	0.3384615384615385	0.0	0.0
Pde2a	1	0.6	0.0	0.0

Supplementary Table S2

The primer sequences for QPCR.

Gene	Forward (5’-3’)	Reverse (5’-3’)
β-actin	TCTGGCATCACACCTTCTACAAT	TGTTGGCTTTGGGATTCAGG
elavl3	AGACAAGATCACAGGCCAGAGCTT	TGGTCTGCAGTTTGAGACCGTTGA
mbp	AATCAGCAGGTTCTTCGGAGGAGA	AAGAAATGCACGACAGGGTTGACG
nrd	TGCAGAGTTGCACAATTCCC	GGTTTGAGCAGACTAATTCAGGTTC
gap43	TGCTGCATCAGAAGAACTAA	CCTCCGGTTTGATTCCATC
nrf2	CCAACTACTCCCAGGTTGCCC	GTGACTGAAACGTAGCCGAAGA
cat	AGGGCAACTGGGATCTTACA	TTTATGGGACCAGACCTTGG
cu/zn-sod	GTCGTCTGGCTTGTGGAGTG	TGTCAGCGGGCTAGTGCTT
mn-sod	AATCAGCAGGTTCTTCGGAGGAGA	ACACTCGGTTGCTCTCTTTTCTCT
kdr	GCCTGATCCACAACTGCTTCC	CTCTCTCACACGACTCAATGC
atp2a1	TCCGTGGTGCCGTCTACTACTTC	CCAGAGCCAAGCAGGTTGTGATG
cacnalab	CAGCCGCCACAATCCACAGAC	TGACACCACCCGTTCCAGAGAG
ppp3ca	AGCAGCATCAGCACCATCATCATC	GGCGAATGTGTTTCTCCCTCCTC
p53	GGGCAATCAGCGAGCAAA	ACTGACCTTCCTGAGTCTCCA
caspase3	CCGCTGCCCATCACTA	ATCCTTTCACGACCATCT
caspase 9	AAATACATAGCAAGGCAACC	CACAGGGAATCAAGAAAGG
bax	GGCTATTTCAACCAGGGTTCC	TGCGAATCACCAATGCTGT

Supplementary Table S3

The acute toxicity results following exposure to MK are as follows (data presented as survival rate; the experiment was conducted with three biological replicates, each containing 10 eggs per well):

Acute toxicity results from exposure to MK

	0			50 μg/L			100 μg/L			200 μg/L			400 μg/L			800 μg/L			1600 μg/L		
24 hpf	10	10	10	10	10	10	10	10	10	10	10	10	10	10	10	9	10	10	8	9	8
48 hpf	10	10	9	10	10	9	9	10	10	9	8	8	8	8	8	7	8	8	6	8	7
72 hpf	9	10	9	10	10	9	9	8	8	6	7	7	4	6	5	4	5	5	4	4	3
96 hpf	9	10	9	9	10	9	7	8	8	4	6	5	3	2	2	1	1	2	0	0	0

Figure S1

Guidelines for the care and use of laboratory animals

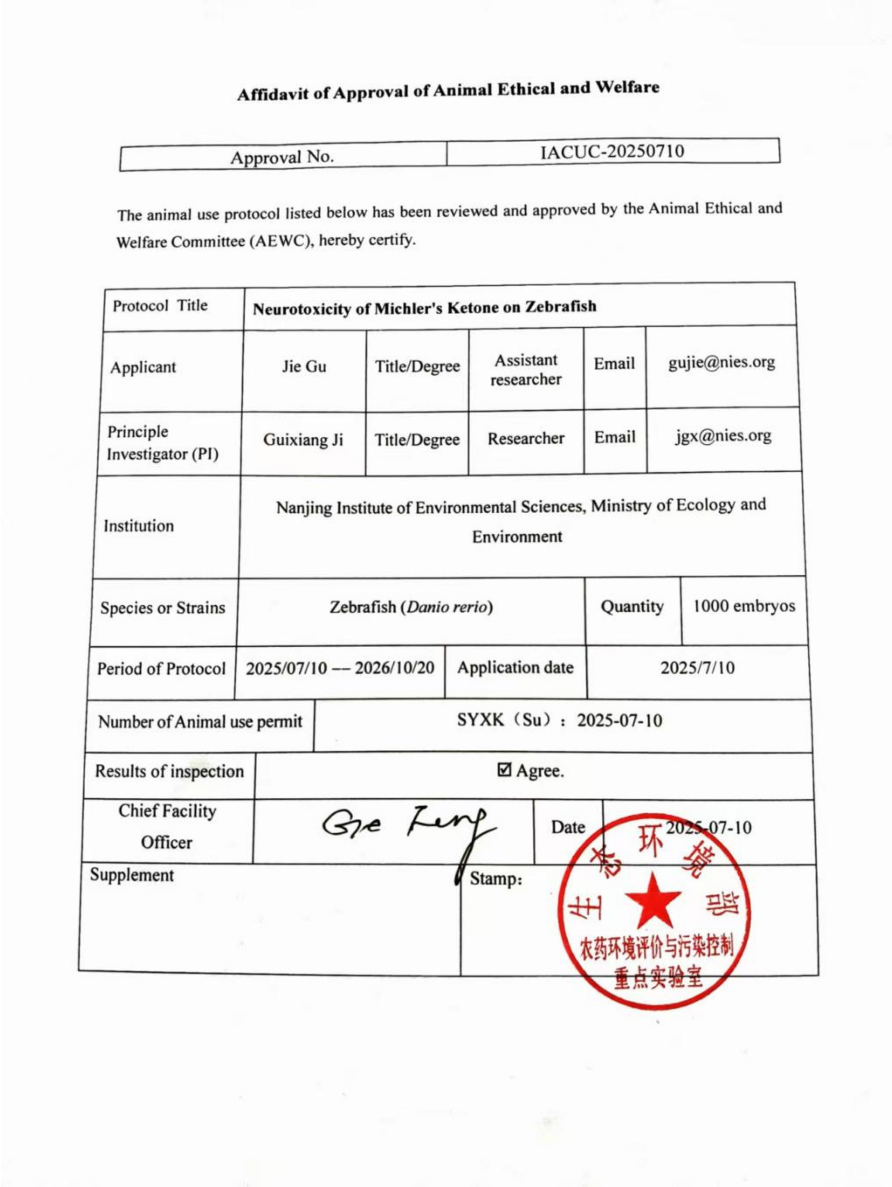

Affidavit of Approval of Animal Ethical and Welfare

Approval No.	IACUC-20250710

The animal use protocol listed below has been reviewed and approved by the Animal Ethical and Welfare Committee (AEWC), hereby certify.

Protocol Title	**Neurotoxicity of Michler's Ketone on Zebrafish**				
Applicant	Jie Gu	Title/Degree	Assistant researcher	Email	gujie@nies.org
Principle Investigator (PI)	Guixiang Ji	Title/Degree	Researcher	Email	jgx@nies.org
Institution	Nanjing Institute of Environmental Sciences, Ministry of Ecology and Environment				
Species or Strains	Zebrafish (*Danio rerio*)			Quantity	1000 embryos
Period of Protocol	2025/07/10 –– 2026/10/20	Application date	2025/7/10		
Number of Animal use permit	SYXK（Su）：2025-07-10				
Results of inspection	☑ Agree.				
Chief Facility Officer	Ge Feng	Date	2025-07-10		
Supplement		Stamp:	生态环境部 农药环境评价与污染控制重点实验室		

Figure S2

Region for fluorescence intensity quantification (*Tg(huc:eGFP)* embryo)

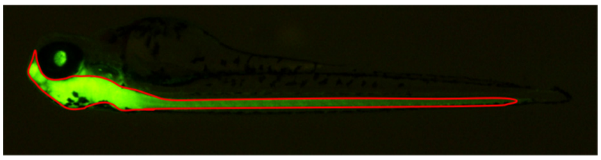

Supplement: Supplementary file 1 [file biology-15-00003-s001.zip › biology-3998682-supplementary.pdf]
